# Paper-Based Microfluidic Platforms for Understanding the Role of Exosomes in the Pathogenesis of Major Blindness-Threatening Diseases

**DOI:** 10.3390/nano8050310

**Published:** 2018-05-08

**Authors:** Min-Yen Hsu, Chun-Chih Chiu, Juan-Yuan Wang, Chin-Te Huang, Yu-Fang Huang, Jyh-Cheng Liou, Chihchen Chen, Hung-Chi Chen, Chao-Min Cheng

**Affiliations:** 1 Department of Ophthalmology, Chung Shan Medical University Hospital, Taichung 402, Taiwan; my.scott.hsu@gmail.com (M.-Y.H.); liou.outcast@gmail.com (J.-C.L.); 2 School of Medicine, Chung Shan Medical University, Taichung 402, Taiwan; 3 Department of Internal Medicine, National Taiwan University Hospital, Taipei 100, Taiwan; hank011028@gmail.com; 4 Department of Ophthalmology, Taichung Veterans General Hospital, Taichung 407, Taiwan; cywang0926@yahoo.com.tw (J.-Y.W.); i5496604@gmail.com (Y.-F.H.); 5 Institute of Medical Sciences, Tzu-Chi University, Hualien 970, Taiwan; chintehuang@hotmail.com; 6 Department of Optometry, Chung Shan Medical University, Taichung 407, Taiwan; 7 Institute of Medicine, Chung Shan Medical University, Taichung 407, Taiwan; 8 Institute of Nanoengineering and Microsystems, National Tsing Hua University, Hsinchu 300, Taiwan; chihchen23@gmail.com; 9 Department of Ophthalmology and Department of Medicine, Chang Gung Memorial Hospital and Chang Gung University College of Medicine, Linkou 244 and Taoyuan 333, Taiwan; 10 Institute of Biomedical Engineering, National Tsing Hua University, Hsinchu 300, Taiwan

**Keywords:** Glaucoma, age-related macular degeneration, exosomes, microfluidics, lab on a chip

## Abstract

Emerging roles of exosomes in the pathogenesis of major blindness-threatening diseases, such as age-related macular degeneration, glaucoma, and corneal dystrophy, were discovered by aqueous humor analysis. A new diagnostic method using cellulose-based devices and microfluidic chip techniques for the isolation of exosomes from aqueous humor is less cumbersome and saves time. This method will enable more investigations for aqueous humor analysis in the future.

## 1. Introduction

Exosomes, the extracellular membrane vesicles secreted via cell exocytosis, were first discovered in 1983 by biochemists Pan and Johnstone while researching transferrin receptors during the maturation of sheep reticulocytes [1]. However, the roles of exosomes in physiology and disease were not fully elucidated until recent decades.

Exosomes are approximately 30- to 120-nm-sized particles that contain lipids, proteins, and small fragments of RNA or microRNA [2]. During circulation, they may be endocytosed into another host cell, where they induce RNA integration, which contributes to intercellular signal transduction. Exosomes have been shown to play an important role in several diseases, such as cancer metastasis and immune disorders, which both heavily rely on intercellular communication [3].

The formation and release of exosomes is part of the normal endosomal delivery system present in almost all cells of the human body. Acidification occurs in the early phase of endosome formation. The acidification process sorts endosomal contents into three separate pathways. One pathway recycles contents back to the cellular surface. Another pathway transfers contents to lysosomes for degradation. The last pathway forms late endosomes containing many small intra-luminal vesicles (ILVs). When this occurs, the late endosome is called a multivesicular body (MVB). MVBs can be degraded after fusing with the lysosome. Otherwise, MVBs can be transferred to the cell surface, where they fuse with the plasma membrane and release their cellular contents into the extracellular space. These particular late endosomes are termed exosomes [4].

Exosomes can easily be extracted from small volumes of bodily fluids, including serum, urine, cerebrospinal fluid, and aqueous humor. Because of their presence in nearly all cells of the human body and their recently identified roles in the pathophysiology of several diseases, exosomes are being increasingly investigated for their potential contributions to in vivo diagnostics (IVD) [5,6,7,8].

## 2. Role of Exosomes in Ophthalmological Diseases

Currently, glaucoma and age-related macular degeneration (AMD) are the second and third leading causes of blindness worldwide after cataracts. According to 2010 WHO global data on visual impairment, glaucoma and AMD account for a combined 13% (AMD: 5%, glaucoma: 8%) of all cases of blindness [9]. Research has shown that the intraocular exosome-related pathway plays a significant role in the pathophysiology of both diseases [10,11]. By investigating intraocular exosomes, it might be possible to develop a new diagnostic approach using aqueous humor sampling that better manages these two globally dominant blindness-causing diseases.

## 3. Role of Exosomes in Aqueous Humor Homeostasis and Glaucoma

**Figure 1:**
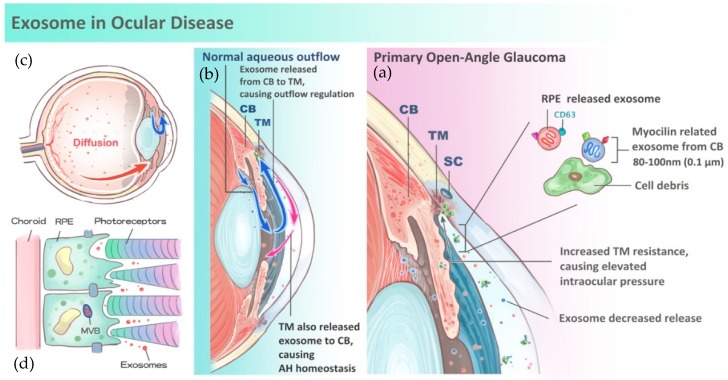
Schematic diagram of exosomes in ocular disease. (**a**) Exosomes (Red dot) are 80 to 100-nm-wide vesicles that can be released from the retina and diffused anteriorly into the aqueous humor (red arrow). Exosomes can also be released from the ciliary body to the trabecular meshwork (Blue arrow). (**b**) In normal aqueous flow conditions, exosomes are released from the ciliary body (CB) to the trabecular meshwork (TM) and send signals to change resistance in the trabecular meshwork, which controls aqueous outflow (blue arrows). Then, trabecular meshwork (TM) may release exosomes back to the ciliary body (red arrows), which produces aqueous humor. In this mechanism, aqueous humor homeostasis can be maintained, which contributes to intraocular pressure. (**c**) In patients with primary open angle glaucoma, Myocilin-related exosomes (blue dots) are released from the ciliary body. Myocilin mutations disrupt aqueous humor homeostasis. Thus, more debris will block the trabecular meshwork and cause an elevation in intraocular pressure, which may cause glaucomatous optic neuropathy and future loss of visual acuity. (**d**) Exosomes (Red dot) released from the retinal pigment epithelium (RPE) to surrounding RPE cells may cause further autophagy of retinal cells and lead to the formation of drusen, which largely contributes to the formation of age-related macular degeneration. Exosomes released by RPE can be diffused into the anterior chamber (Red arrows).

The aqueous humor is a clear fluid circulating between the posterior and anterior chambers of the human eye that maintains intraocular pressure (IOP), among other things. The aqueous humor is a critical component in the pathophysiology of glaucoma development. Aqueous humor is produced from plasma via the epithelium of the ciliary body pars plicata. Circulating aqueous humor flows around the lens, through the pupil, and into the anterior chamber. There are two main routes of aqueous outflow into systemic circulation: trabecular meshwork, which accounts for approximately 90% of flow; and uveoscleral outflow, which accounts for the remaining 10%. Exosomes in aqueous humor may contribute to intercellular communication in the eye [12]. The ciliary body releases exosomes and presents translational signals to the trabecular meshwork using encapsulated RNA fragments [13]. Furthermore, exosomes are also secreted from the trabecular meshwork and travel back to the ciliary body. Through this mutual signal transduction and modification, homeostasis of aqueous humor, i.e., normal IOP, is maintained (Figure 1a,b).

In addition to delivering vital translational signals, exosomes also manage aqueous humor via cellular contents. Myocilin, a protein mainly secreted in its soluble form in trabecular cell-conditioned media and fresh eye samples, is believed to significantly influence the disease progression of glaucoma (Figure 1b) [9,10,13]. Myocilin is abundant in aqueous humor and is bound to exosomes. Myocilin serves as a cell debris scavenger within the trabecular meshwork to keep it clean. Mutations in myo-C, the gene encoding myocilin, were identified in some cases of primary open angle glaucoma (POAG) [14] (Figure 1c). POAG patients are prone to developing glaucoma at an early age with markedly elevated IOP.

Mutated myo-C may disrupt other protein-protein interactions and interfere with aqueous humor homeostasis [15]. A lack of myocilin causes obstruction of the trabecular meshwork and aqueous humor cannot be drained out, which eventually results in elevated IOP [16]. The role of myocilin in the exosome pathway should be clarified in future research.

Extraordinarily elevated IOP damages the nerve fiber layer of the optic disc resulting in irreversible visual field loss. Glaucoma is known as a silent vision killer because no specific biomarkers can be applied as a reliable predictive factor. To date, only IOP measurement, visual field tests, and optical coherence tomography (OCT) for disc retinal nerve fiber thickness can be used for diagnosing glaucoma [17]. However, none of these approaches are sufficiently accurate, and they are time-consuming and highly instrument-dependent.

Topical steroids are widely used as anti-inflammatory medication in ophthalmology. Approximately 20% of patients with long-term steroid use develop glaucoma. It has been reported that steroid use results in glaucoma due to induced fluctuations in aqueous exosome levels [13,14]. Thus, periodic monitoring of steroid-using patients seems crucial to their treatment.

In summary, our increased understanding of how exosomes contribute to the development of glaucoma prompts consideration of in vitro exosome measurement from aqueous humor as a feasible and effective alternative to existing diagnostic methods. This methodology is effective, easy to perform, and can be applied to primary diagnosis as well as monitoring for adverse effects of long-term therapeutic steroid use.

## 4. Role of Exosomes in Age-Related Macular Degeneration

The retina is the most delicate structure in the human eye. It is a light-sensitive neural tissue that acts like film in a camera. The retina consists of three basic cells type: photoreceptors, neuronal cells, and glial cells. These cells construct the ten layers of the retina. The retinal layers begin with the inner-most nerve fiber layer, progress through the inner plexiform layer, and end in the outer-most layer of retinal pigment epithelium (RPE) and Burch membrane, which is firmly attached to the choroid. RPE cells have tight junctional complexes that act as an outer blood-retinal barrier, and they prevent extracellular fluid from leaking into the subretinal space while actively pumping water out. The macula lies in the center of the retina and is the region of sharpest visual acuity. The macula contains multiple layers of ganglion cells while the peripheral retina contains only a single layer [18].

Age-related macular degeneration (AMD), a progressive chronic disease of the central retina, is the leading cause of blindness among populations aged 65 years or older in industrialized nations. According to a global systematic literature review from The Lancet Global Health in 2014, AMD occurs in 8.69% of the global population [19]. Furthermore, 196 million people are projected to contract the disease by 2020, and this number is expected to increase to 288 million in 2040 as the average life expectancy slowly increases.

Patients with early-stage AMD are typically asymptomatic. Yellowish drusen, which is essentially cellular trash depositions, is observable beneath the retinal pigment epithelium in early-stage AMD. This drusen interferes with the normal retinal blood supply, which leads to photoreceptor death. Rapid deterioration of central vision often presents with an increased loss of photoreceptors and neovascularization over the macular area. Macular neovascularization is often referred to as “wet” AMD, and a lack of neovascularization is referred to as “dry” AMD. People with wet AMD complain of deceased visual acuity, a positive central scotoma, image distortion, and changes in observed object size [20].

Current treatment for AMD is limited because the causal molecular pathways are not understood. Nutrition adjustment and corrective lenses are used as treatments in early cases. Laser coagulation, photodynamic therapy, anti-vascular endothelial growth factor (anti-VEGF) administration, and visual rehabilitation are used in advanced cases [9,21,22].

Exosomes are believed to play a crucial role in intercellular communication, especially in AMD. Exosomes can diffuse anteriorly from the vitreous to the aqueous humor and can be detected through many methods (Figure 1a). Similar to their role in the development of glaucoma, they also play a role in AMD development. Previous research found that upregulation of intercellular protein release via exosomes was observed in drusen formation, which is believed to be relevant to the pathophysiology of AMD [21]. Furthermore, current studies have discovered exosome release from retinal pigment epithelium (RPE) cell lines in vitro and noted that associated exosome activity is altered in AMD cases [7,22]. It is speculated that these alterations, which may be caused by genetic mutations, contribute to AMD by affecting communication between the RPE and retinal photoreceptors [23] (Figure 1d). It was also suggested that gradual autophagy of the surrounding RPE was due to intercellular communication through exosomes. Isolation of exosomes in patients with retinopathy using aqueous sampling proved to be less cumbersome and less volume-demanding than other methods [24,25].

## 5. Perspectives—Current Research Limitations and Potential Breakthrough Using In Vitro Diagnostic Tools

The distribution and concentration of many biological factors, such as cytokines, pathogens, and exosomes, are more different in the eye than in other bodily fluids. These materials are scarcer in the eye because of the blood-retinal barrier. For this reason, the exosomes isolated from aqueous humor are mostly secreted by intraocular tissues. This feature increases the effectiveness and feasibility of aqueous humor analyses to investigate and diagnose eye diseases [23,24,25,26].

We duly note that current techniques for exosome isolation from bodily fluid require repetitive ultra-centrifugations, which contribute to the time and fluid volume requirements. Because only 100–200 µL of aqueous humor can be obtained from a single collection, exosome isolation from this material has some obstacles to overcome.

**Figure 2:**
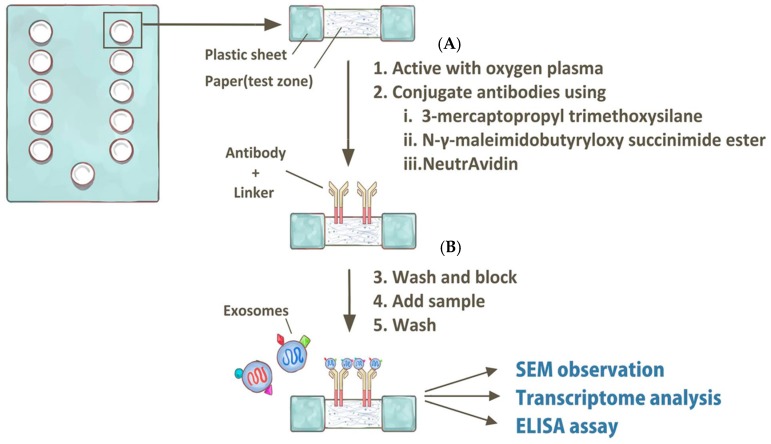
Schematic protocol of the paper-based assay for isolation of exosomes within aqueous humor. (**A**) The surface of the paper sheet test zone was activated with a brief treatment with oxygen plasma and then conjugated to capture molecules using 3-mercaptopropyl trimethoxysilane, *N*-γ-maleimidobutyryloxy succinimide ester, and NeutrAvidin. (**B**) Then, we washed and blocked the test zone, added 25 µL of aqueous humor, and finished with a wash. Exosomes isolated on this functionalized paper surface could be further characterized by scanning electron microscopy (SEM), transcriptome analysis (by microfluidic chip), and ELISA assays.

**Figure 3:**
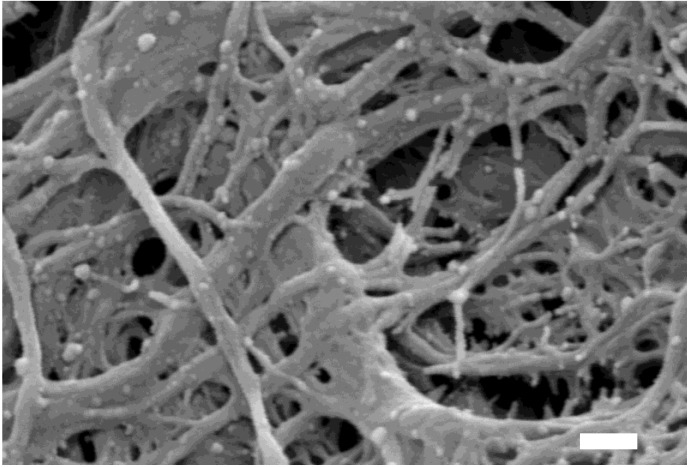
Scanning electron microscopy image of exosomes in aqueous humor samples captured on microfluidic filter paper. Isolated CD63+ exosomes in aqueous humor from patients with age-related macular degenerations. White scale bar = 200 micrometers.

Recently, several reports have been published regarding a method for exosome isolation from aqueous humor using a paper-based immunoaffinity assay, a scanning electron microscope, and a micro-fluidic RNA detecting chip (Agilent 2100 bioanalyzer) [27,28]. A detailed protocol for this method is shown in Figure 2. This novel isolation method has some advantages in aqueous humor analysis over previous methods: it requires only 25 µL of sample and eliminates the repetitive ultra-centrifugations, which reduces the analysis time from at least 5 h to only 1 h. Exosomes isolated with this functionalized paper surface can be further characterized by scanning electron microscopy (Figure 3). In the literature review, one article mentioned a simple sandwich immunofluorescence assay (sIFA) microfluidic device for aqueous humor detection [29]. However, this PDMS-based microfluidic device demanded delicate design and fabrication of UV-masking to create the microstructure. Thus, these protocols of fabrication made the PDMS-based microfluidic device for aqueous humor detection cumbersome. Besides, the advantages of paper microfluidics are well established. Modification of paper with silane is published and shows practically relevant limit of detections(LODs) with high expandability and adaptability [30].

Using microfluidic paper for exosome isolation from aqueous humor for diagnostics can be applied in the future for personalized medicine [31]. The therapeutic response to certain treatments, status of disease, or subtype of disease can be monitored more easily with this paper-based microfluidic platform.

For intraocular samples, such as aqueous humor, related point-of-care diagnostic methods have not yet been developed due to technical limitations and insufficient understanding of the molecular disease pathways. Further study of the clinical value of exosome isolation for diagnosis and treatment of glaucoma and AMD, as well as implementation of new and novel exosome isolation techniques, will broaden the scope and impact of in vitro investigation for understanding pathways underlying these two major causes of blindness.
